# The effect of 2,5-di-(tert-butyl)-1,4-benzohydroquinone (TBQ) on intracellular Ca^2+^ handling in rat ventricular myocytes

**DOI:** 10.1016/j.ceca.2015.05.002

**Published:** 2015-08-01

**Authors:** L. Miller, D.J. Greensmith, R. Sankaranarayanan, S.C. O’Neill, D.A. Eisner

**Affiliations:** a Unit of Cardiac Physiology, Institute of Cardiovascular Sciences, 3.06 Core Technology Facility, 46 Grafton Street, Manchester M13 9NT, United Kingdom; b Biomedical Research Centre, School of Environment and Life Sciences, University of Salford, G.35 Peel Building, Salford M5 4WT, United Kingdom

**Keywords:** TBQ, SERCA, Calcium, Myocyte

## Abstract

2,5-Di-(tert-butyl)-1,4-benzohydroquinone (TBQ) is a reversible inhibitor of SERCA, potentially making it a useful tool to study the effects of SERCA inhibition in cardiac cells. However, it is unknown if TBQ also has effects on other components of ventricular Ca handling. The aim of these experiments was to characterise the effects of TBQ on Ca handling in rat ventricular myocytes and assess its suitability as a specific inhibitor of SERCA. This was achieved by voltage clamp via perforated patch and [Ca^2+^]_*i*_ measurement using Fluo-3 AM. TBQ produced a fully reversible, concentration dependent decrease in the rate of systolic Ca decay. 10 μM TBQ decreased the amplitude of the systolic Ca transient by 48 ± 5% and the rate of decay by 54 ± 6%. SR Ca content was also reduced by 62 ± 4%. However, 10 μM TBQ also decreased the peak L-type Ca current by 23 ± 7%. At higher concentrations (100 μM), TBQ also activated an outward current with a current–voltage relationship consistent with a potassium current. This outward current was abolished by Glibenclamide (100 μM). These data show that TBQ can be used to reversibly inhibit SERCA. However, at concentrations that decrease SERCA activity, TBQ also decreases the L-type Ca current and (at higher concentrations) activates an outward current which appears to be an ATP dependent potassium current. We conclude that TBQ cannot be used as a specific inhibitor of SERCA in rat ventricular myocytes.

## Highlights

• The suitability of TBQ as a specific inhibitor of SERCA was investigated.
• TBQ decreased SERCA activity in a concentration dependent manner.
• TBQ inhibited the calcium current.
• TBQ activated an outward current consistent with an ATP-dependent potassium channel.
• TBQ cannot be used as a specific inhibitor of SERCA in rat cardiac myocytes.

## Graphical abstract

**Figure:**
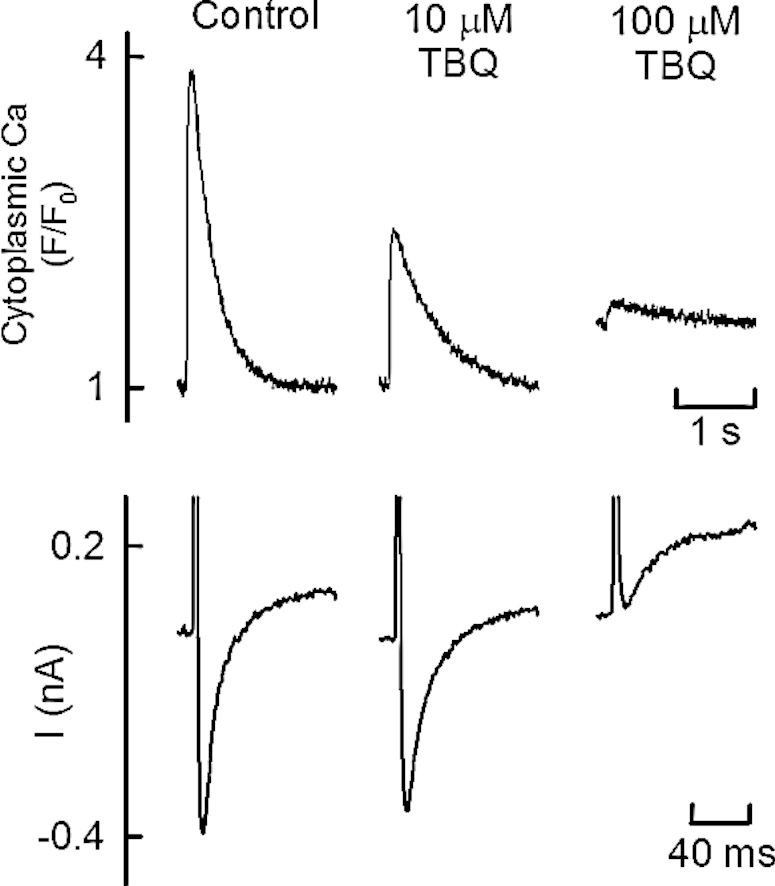

## 1 Introduction

For the heart to function as a pump, the myocardium must relax following systole. At the cell level, this is achieved by removal of systolic Ca from the cytosol restoring diastolic Ca levels. In mammals, the majority of Ca removal occurs by reuptake of Ca into the sarcoplasmic reticulum (SR) by the sarco-endoplasmic reticulum Ca ATPase (SERCA) [1]. In rat cardiac myocytes, SERCA uptake accounts for around 90% of the total cytosolic Ca removal, with the remainder due to the Na–Ca exchange (NCX) and the plasma membrane Ca-ATPase (PMCA) [2].

During conditions such as metabolic inhibition [3] and heart disease [4], both contraction and relaxation may be affected. Decreased relaxation (negative lusitropy) has been attributed to impaired SERCA function and thence a slowing of systolic Ca removal [4,5]. Studying the role of SERCA requires, ideally, a selective and reversible inhibitor. Thapsigargin has been used extensively [6–8] but its actions are irreversible. Another inhibitor is 2,5-di-(tert-butyl)-1,4-benzohydroquinone (TBQ). This is reversible and has been used previously in many cell types including ventricular myocytes and muscle preparations [9,10]. However, it is currently unknown whether TBQ has an effect on other Ca handling components of ventricular myocytes. The aim of the present study was to characterise the effects of TBQ on intracellular Ca handling in rat ventricular myocytes and to assess the suitability of TBQ as a specific inhibitor of SERCA.

We demonstrate that, in addition to inhibiting SERCA, TBQ decreases *I*_Ca-L_ and activates an outward potassium current leaving its suitability as a specific SERCA inhibitor in question.

## 2 Methods

### 2.1 Isolation of ventricular myocytes

Rat ventricular myocytes were isolated by enzymatic digestion. Male Wistar rats (weighing approximately 200 g) were killed in accordance with the Animals (Scientific Procedures) Act 1982. Hearts were quickly excised and mounted on a Langendorff apparatus by aortic cannulation to allow retrograde perfusion of the coronary arteries. The heart was perfused with a calcium free solution containing (in mM) NaCl; 134, HEPES; 10, Glucose; 11.1, NaH_2_PO_4_; 1.2, MgSO_4_; 1.2, KCl; 4 (titrated to pH 7.34 at room temperature). Following a 5 min wash with Ca free solution, Collagenase (Worthington Biochemical Cooperation, NJ, USA) and protease (Sigma–Aldrich, Dorset, UK) were added to the perfusate at concentrations of 95.4 units/mg and 0.1 units/mg respectively for 7 min. The ventricles were then removed and minced in a Taurine solution containing (in mM) NaCl; 115, HEPES; 10, Glucose; 11.1, NaH_2_PO_4_; 1.2, MgSO_4_; 1.2, KCl; 4, Taurine; 50 (titrated to pH 7.34 at room temperature). Gentle agitation was used to release single myocytes. Cells were stored in Taurine solution at room temperature prior to use.

### 2.2 Electrophysiological recordings

Myocytes were voltage clamped using the perforated patch technique (Axon Instruments, CA, USA). Microelectrodes (resistance 4–5 MΩ) were filled with solution consisting of (in mM); 10 NaCl; 20 KCl; 10 HEPES; 125 K_3_CH_3_O_3_S; 5 MgCl_2_; 0.1 K_2_EGTA (titrated to pH 7.2 using KOH) and 240 μg/ml amphotericin B. Unless otherwise stated, cells were stimulated with a 100 ms duration voltage step from a holding potential of −40 mV to 0 mV, at a frequency of 0.5 Hz using pClamp software (Axon Instruments, CA, USA). Cells were bathed in a standard experimental solution (134 mM NaCl, 10 HEPES, 11.1 Glucose, 1.2 MgCl_2_, 4 KCl, 1 CaCl_2_, titrated to pH 7.34 at room temperature). Most experiments were performed at 37 °C. TBQ (Sigma–Aldrich, Dorset, UK) was applied at final concentrations of 1, 3, 10 and 100 μM from a 100 mM stock in ethanol. Control experiments (data not shown) demonstrated that 0.1% ethanol (the highest concentration applied to cells) had no observable effect on any measured parameter.

### 2.3 Measurement of intracellular Ca and SR Ca content

Myocytes were loaded with the membrane permeable acetoxymethyl (AM) ester form of the fluorescent Ca indicator Fluo-3 (5 μmol/L for 10 min). Fluo-3 was excited at 488 nm and emitted fluorescence measured with a 515 nm long pass filter. Background fluorescence was subtracted from all signals. Changes in [Ca^2+^]_*i*_ are represented by changes in fluorescence expressed as: *F/F*_0_, where *F*_0_ is control, diastolic fluorescence. SR Ca content was measured by rapid application of 10 mM caffeine and integration of the resulting inward NCX current as described previously in detail [11].

### 2.4 Data analysis and statistics

Data were analysed with custom written Excel routines [12]. Results are expressed as mean data ± SEM for *n* cells. Statistical significance (*p* < 0.05) was determined using *t*-tests and repeated measures ANOVAs.

## 3 Results

**Fig. 1:**
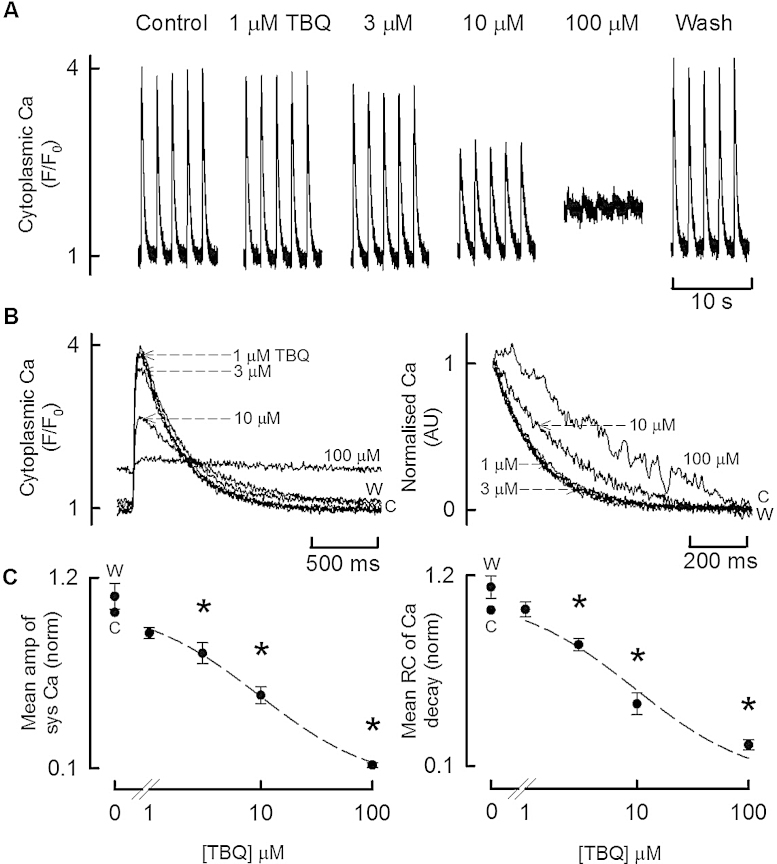
The effects of TBQ on the Ca transient. (A) Specimen [Ca^2+^]*_i_* transients measured with Fluo-3. The cell was stimulated at 0.5 Hz with a 100 ms duration depolarising pulse from −40 to 0 mV. TBQ was applied in the concentrations shown. (B) Expanded time base overlays showing averaged transients from (A). Left shows original records and right normalised. (C) Average data (*n* = 16 cells from 8 animals) showing (left) the amplitude of systolic Ca and (right) rate constant of decay of systolic Ca. All data have been normalised to the values before addition of TBQ (“C”). “W” denotes values after removing the final concentration of TBQ (*p < 0.05 vs. control).

The experiment illustrated in Fig. 1 (representative of data from 16 cells) shows the effect of TBQ on Ca transients evoked by depolarising pulses. TBQ produced a concentration dependent decrease in the amplitude of systolic Ca and rate of systolic Ca decay and an increase of diastolic Ca (Fig. 1A). The effects on the kinetics of the Ca transient can be seen more clearly in the fast time base records of Fig. 1B. Average data from these experiments are presented in Fig. 1C.

**Fig. 2:**
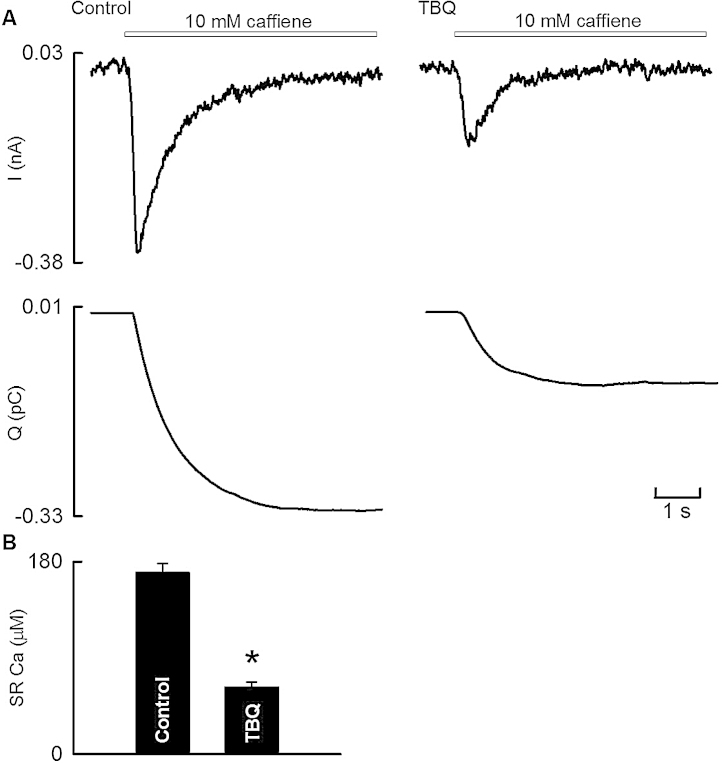
The effect of TBQ on SR Ca content. (A) Specimen *I*_NCX_ records (top panel) and corresponding integrals (lower panel) in control (left) and following 10 μM TBQ (right) evoked by rapid application of 10 mM caffeine (white bar). (B) Average SR Ca (*n* = 5 cells from 3 animals, *p < 0.05 vs. control).

10 μM TBQ was used to study the effect on SR Ca content. This concentration was used as it decreased SERCA activity without markedly reducing the systolic Ca transient. Fig. 2A (representative of 5 cells) shows specimen NCX currents (top) produced by rapid application of 10 mM caffeine in control and following TBQ and the integrals of those currents (below). TBQ consistently decreased this integral. On average (Fig. 2B) TBQ decreased SR Ca content by 62 ± 4%.

**Fig. 3:**
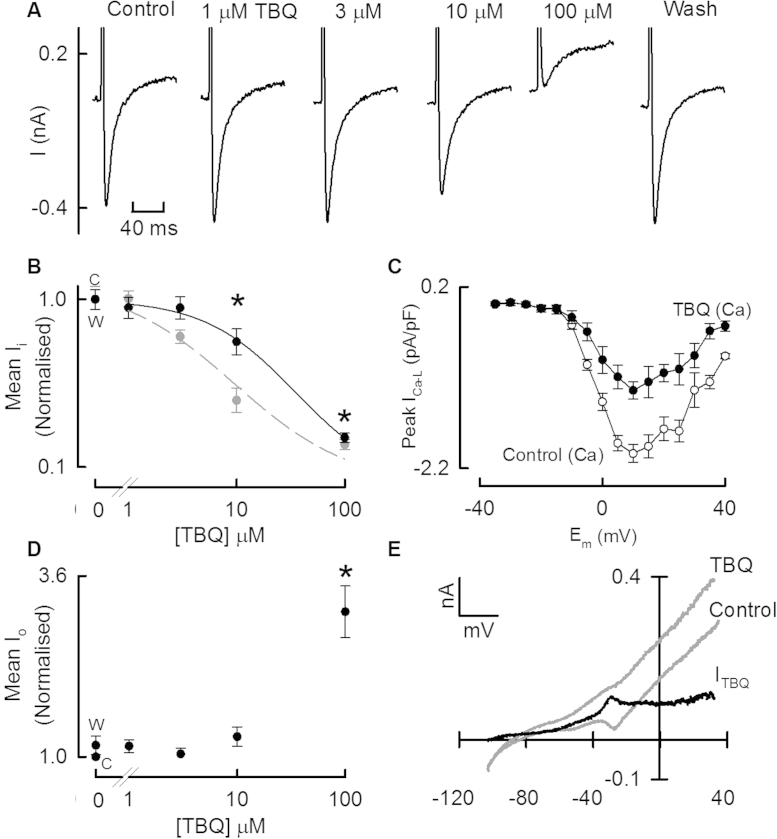
The effects of TBQ on sarcolemmal currents. (A) Specimen membrane currents in response to a 100 ms depolarising pulse from −40 to 0 mV. Panels show (left to right) control, increasing concentrations of TBQ and washout. (B) Average data (*n* = 17 cells from 8 animals) showing peak inward current (black). The effect on the rate constant of systolic calcium removal is superimposed for reference (grey and dashed curve). (C) *I*_Ca-L_*I*–*V* plot in control (open symbols) and presence of 10 μM TBQ (filled symbols). (D) Average data shows the peak outward component of the currents represented by (A). (E) Specimen IV relationship produced by a voltage ramp protocol (−120 to 40 mV) showing the control current, the current after exposure to TBQ and the TBQ sensitive current determined by subtraction of TBQ from control (*I*_TBQ_). This record is typical of 5 experiments. *p < 0.05 vs. control.

In these experiments we also found that application of TBQ had marked effects on membrane current. The current records illustrated in Fig. 3A were recorded at the same time as the Ca transients in Fig. 1. Application of TBQ produced a concentration dependent decrease of peak *I*_Ca-L_ (mean data shown as black trace in Fig. 3B). Fig. 3C shows the effect of 10 μM TBQ on the mean *I*–*V* relationship of *I*_Ca-L_. TBQ decreased *I*_Ca-L_ amplitude over the entire voltage range tested but did not shift the IV relationship. At higher concentrations (100 μM, Fig. 3A), application of TBQ also resulted in the appearance of an outward current (see also average data in Fig. 3D). In order to study the outward current in more detail, in some experiments we applied a depolarising voltage ramp. Fig. 3E shows the resulting current under control conditions and following exposure to 100 μM TBQ. Subtraction of the control current from that produced by TBQ gives the TBQ sensitive current. This experiment was typical of 5 cells.

**Fig. 4:**
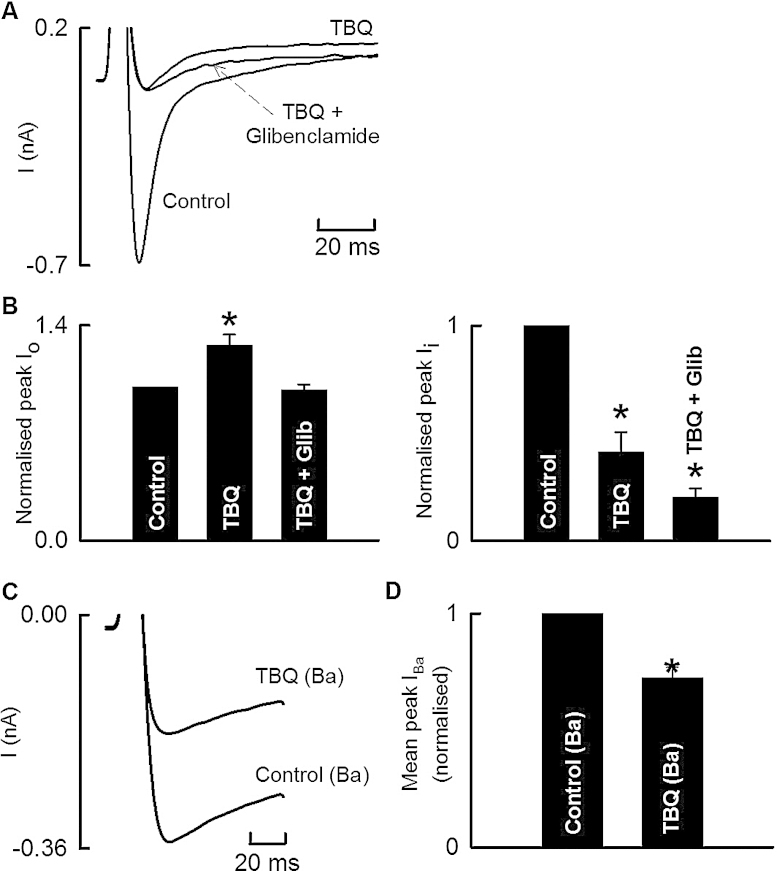
Characterisation of sarcolemmal currents produced by TBQ. (A) Specimen current records. The cell was stimulated at 0.5 Hz with a 100 ms duration depolarising pulse from −40 to 0 mV. Currents were recorded in control then exposed to 100 μM TBQ. TBQ was then washed off and 100 μM Glibenclamide added before adding 100 μM TBQ with 100 μM Glibenclamide. (B) Average data (*n* = 4 cells from 3 animals) showing (left) the amplitude of the outward component and (right) the amplitude of the inward component. (C) Specimen *I*_Ba_ records in control and following 10 μM TBQ. (D) Average peak *I*_Ba_ data (*n* = 4 cells from 4 animals) (*p < 0.05 vs. control).

One concern with the interpretation of the results of Fig. 3A–C is the possibility that, at least at higher concentrations of TBQ, the apparent decrease of inward current resulted from the increase of outward current. We discovered that the outward current could be inhibited by Glibenclamide (100 μM). The specimen current records of Fig. 4A were recorded from a cell where 100 μM TBQ was applied to replicate the effect observed in the experiment of Fig. 3. Again, TBQ decreased the inward current and increased the outward. Subsequently, TBQ was removed, and 100 μM Glibenclamide applied. 100 μM TBQ was then re-applied (in the maintained presence of 100 μM Glibenclamide). In the presence of Glibenclamide, the reduction of *I*_Ca-L_ produced by TBQ persisted; however, the activation of the outward component was prevented. Glibenclamide alone produced no effect on the calcium current (0.44 ± 0.09 vs. 0.48 ± 0.14 nA, *n* = 5, *p* > 0.05; data not shown). Average data for 4 cells are shown in Fig. 4B. The experiments of Fig. 4C were carried out to determine if 10 μM TBQ had a *direct* effect on the L-type Ca current or, alternatively, whether the effects were an indirect consequence of changes of cellular Ca handling resulting from inhibition of SERCA. 10 μM TBQ was chosen for the reasons stated above. Fig. 4C shows a current trace recorded from a cell where Ca in the external solution had been replaced by Ba. On average (Fig. 4D) peak *I*_Ba_ was reduced by 27.7 ± 4.6%.

**Fig. 5:**
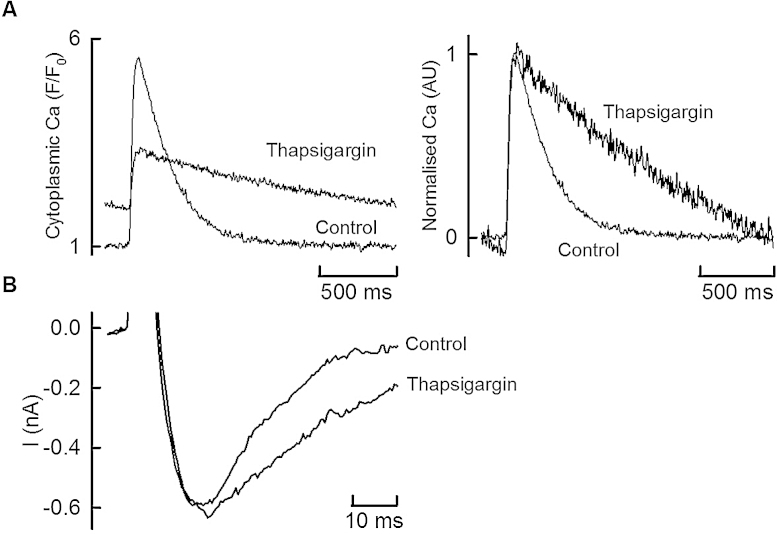
Does SERCA inhibition activate *I*_K-ATP_? (A) Specimen [Ca^2+^]*_i_* transients showing original traces (left panel) and normalised traces (right panel) for direct comparison of the decay phase. 1 μM Thapsigargin was applied to inhibit SERCA. (B) Specimen *I*_Ca-L_ traces in control and following 1 μM Thapsigargin. Application of Thapsigargin was not associated with a decrease in *I*_Ca-L_ or activation of *I*_o_. Representative of 3 cells from 3 animals.

The final question addressed was whether the effects on membrane current were a direct effect of TBQ or, alternatively, secondary to SERCA inhibition. In parallel experiments illustrated in Fig. 5, this was done by examining the effects of 1 μM Thapsigargin, an alternative SERCA inhibitor. Thapsigargin, decreased both the amplitude and rate of decay of systolic Ca to levels comparable to those produced by 100 μM TBQ as shown in Fig. 1 (Ca transient amplitude: TBQ 11.8%, Thapsigargin: 15.1% of control. Rate of systolic Ca decay: TBQ 22.1%, Thapsigargin 8.75% of control). In the presence of Thapsigargin, however, these effects were not associated with a decreased *I*_Ca-L_ or activation of *I*_o_. A consistent slowed *I*_Ca-L_ inactivation was observed, consistent with a decreased systolic Ca.

## 4 Discussion

The original aim of this study was to characterise the effects of TBQ on intracellular Ca handling in rat cardiac myocytes to determine whether TBQ is suitable for use as a specific inhibitor of SERCA. The results show that TBQ decreases the Ca current and activates an outward current; these effects must be born in mind when using this compound.

### 4.1 The effect of TBQ on intracellular calcium

As expected, TBQ produced a reversible and concentration dependent decrease in both the rate of decay of the systolic Ca transient and the SR Ca content; effects consistent with SERCA inhibition. Previously we have shown that in cardiac myocytes exhibiting spontaneous waves of SR Ca release, SERCA inhibition by 100 μM TBQ, produced only a small decrease (to 94% of control) of SR Ca content immediately prior to the onset of a Ca wave [9]. The above reduction of SR Ca content to 38% produced by TBQ, demonstrates that SERCA inhibition in stimulated cells causes a greater disruption to Ca cycling; presumably due to the finite time available for the SR to refill following CICR. TBQ also produced a concentration dependent decrease in systolic Ca and an increase in diastolic Ca, the latter presumably as a result of severe SERCA impairment resulting in incomplete removal of systolic Ca on each beat.

### 4.2 The effect of TBQ on membrane currents

TBQ produced a concentration dependent decrease of peak *I*_Ca-L_ (Fig. 3); this inhibition was seen at all membrane potentials examined (Fig. 3C). When Ca was replaced with Ba, TBQ also decreased peak *I*_Ba_ (Fig. 4C and D) suggesting a direct effect of TBQ on the L-type Ca channel may contribute to the decreased peak *I*_Ca-L_.

As well as decreasing the inward current, TBQ increased the outward current at the end of the depolarising pulse. The current–voltage relationship confirms that TBQ increases an outward current (as opposed to decreasing an inward current). The IV relationship of the TBQ sensitive current (Fig. 3E) was consistent with that of a potassium current. Furthermore, it appeared to be weakly rectifying. One possible candidate for the current may be an ATP dependent potassium current (*I*_K-ATP_) [13]. This would be consistent with the complete inhibition of the outward current by Glibenclamide [14] (Fig. 4). Such an increase of outward current might be a direct effect of TBQ but it should also be noted that TBQ has been shown to decrease intracellular ATP [15] and might thereby increase the current.

It is also important to note that, in the presence of Glibenclamide, although the outward component is inhibited, the decreased *I*_Ca-L_ persists. This confirms that the above effects on *I*_Ca-L_ are a *direct* effect of TBQ, rather than because the net inward current recorded is simply being offset by a positive component.

### 4.3 The mechanism of the effect of TBQ on systolic Ca

Given the various effects of TBQ identified in this paper, it is worthwhile considering their relative contributions to the observed decrease of the Ca transient. One obvious explanation is provided by the measured decrease of SR Ca content. Previous work has shown that the amplitude of the systolic Ca transient is proportional to the third power of SR Ca content [16]. Following application of 10 μM TBQ, SR Ca content fell to 38% of control (Fig. 2). This would predict a reduction of systolic Ca to (0.38)^3^ = 5.5% control. Therefore, this reduction in SR Ca content can more than explain the reduction of systolic Ca to 52% of control produced by 10 μM TBQ. In addition to this, the decrease of L-type Ca current would produce a more modest fall of the Ca transient [17].

### 4.4 Are the effects of TBQ on sarcolemmal currents due to SERCA inhibition?

The decrease of *I*_Ca-L_ and activation of *I*_K-ATP_ observed in the presence of TBQ may have been a consequence of SERCA inhibition rather than a direct effect of TBQ. That this is not the case is demonstrated by Fig. 5. Thapsigargin decreased both the amplitude and rate of decay of systolic Ca to levels comparable to those produced by TBQ. It did not, however, decrease the amplitude of *I*_Ca-L_ or activate an outward current, indicating that these effects are not secondary to SERCA inhibition. Thapsigargin decreased the rate of decay of the L-type Ca current presumably due to decreasing Ca-dependent inactivation.

### 4.5 The suitability of TBQ as a specific SERCA inhibitor

That TBQ produces effects on sarcolemmal currents brings into question whether it is a useful inhibitor of SERCA. Fig. 3B shows an IC_50_ for SERCA inhibition of about 10 μM. This concentration only produces an approx. 25% inhibition of *I*_Ca-L_ (IC_50_ approx. 40 μM) and no measurable *I*_o_. The relatively high potency for SERCA inhibition means TBQ may be useful where partial, reversible inhibition of SERCA is required or where membrane currents are controlled or irrelevant (for example, in permeablised cells). However, concentrations which produce near complete inhibition of SERCA are associated with an approx. 75% reduction in *I*_Ca-L_, and an approx. 3.1 fold increase in *I*_o_. The magnitude of these “unwanted” effects shows that in the rat cardiac myocyte, TBQ is not a *specific* inhibitor of SERCA, and may be of limited practical use.

## 5 Summary

This study confirms that TBQ is a reversible inhibitor of SERCA in rat ventricular myocytes. However, in these cells, at concentrations that reduce SERCA activity, TBQ also decreases peak *I*_Ca-L_ and activates an outward current which we suggest is *I*_K-ATP_. We therefore conclude that TBQ is unsuitable for use as a *specific* reversible inhibitor of SERCA in cardiac myocytes.

## Conflicts of interest

No conflicts of interest declared.
